# Diabetic Rats Induced Using a High-Fat Diet and Low-Dose Streptozotocin Treatment Exhibit Gut Microbiota Dysbiosis and Osteoporotic Bone Pathologies

**DOI:** 10.3390/nu16081220

**Published:** 2024-04-19

**Authors:** Kuo-Chin Huang, Po-Yao Chuang, Tien-Yu Yang, Yao-Hung Tsai, Yen-Yao Li, Shun-Fu Chang

**Affiliations:** 1 School of Medicine, Chang Gung University College of Medicine, Taoyuan City 33302, Taiwan; kc2672@adm.cgmh.org.tw (K.-C.H.); b9102072@cgmh.org.tw (P.-Y.C.); pku70@adm.cgmh.org.tw (T.-Y.Y.); orma2244@adm.cgmh.org.tw (Y.-H.T.); yao.ortho@gmail.com (Y.-Y.L.); 2 Department of Orthopaedic Surgery, Chiayi Chang Gung Memorial Hospital, Chiayi City 61363, Taiwan; 3 Department of Medical Research and Development, Chiayi Chang Gung Memorial Hospital, Chiayi City 61363, Taiwan

**Keywords:** gut microbiota, diabetic bone disease, firmicutes/bacteroidetes ratio, osteoporosis, type 2 diabetes mellitus

## Abstract

Type 2 diabetes mellitus (T2DM) presents a challenge for individuals today, affecting their health and life quality. Besides its known complications, T2DM has been found to contribute to bone/mineral abnormalities, thereby increasing the vulnerability to bone fragility/fractures. However, there is still a need for appropriate diagnostic approaches and targeted medications to address T2DM-associated bone diseases. This study aims to investigate the relationship between changes in gut microbiota, T2DM, and osteoporosis. To explore this, a T2DM rat model was induced by combining a high-fat diet and low-dose streptozotocin treatment. Our findings reveal that T2DM rats have lower bone mass and reduced levels of bone turnover markers compared to control rats. We also observe significant alterations in gut microbiota in T2DM rats, characterized by a higher relative abundance of Firmicutes (F) and Proteobacteria (P), but a lower relative abundance of Bacteroidetes (B) at the phylum level. Further analysis indicates a correlation between the F/B ratio and bone turnover levels, as well as between the B/P ratio and HbA1c levels. Additionally, at the genus level, we observe an inverse correlation in the relative abundance of *Lachnospiraceae*. These findings show promise for the development of new strategies to diagnose and treat T2DM-associated bone diseases.

## 1. Introduction

Currently, diabetes mellitus (DM) is a widespread metabolic disease that significantly affects the health and quality of life of patients. In addition to well-known complications like neuronal and cardiovascular diseases, DM has also been suggested to contribute to bone and mineral abnormalities. These abnormalities may increase the risk of bone fragility and fractures, making daily living and activity more challenging for patients [1]. The direct effects of insulin deficiency or resistance and hyperglycemia on the bone marrow microenvironment (BMM) are believed to play a role in these abnormalities [2,3,4]. Other factors, such as a chronic inflammation triggered by advanced glycation end products (AGEs) of bone matrix proteins, an abnormal production of adipokines and cytokines, impaired bone-muscle interactions, and abnormal bone marrow fat metabolism and content, may also contribute to these abnormalities [5,6]. Observational research and animal studies suggest that individuals with type 2 diabetes (T2DM), the most prevalent form of diabetes, may have decreased bone strength and an increased risk of fractures [6,7,8]. The increasing prevalence of T2DM and associated skeletal complications can be attributed to demographic changes, longer lifespans, and Western diets and lifestyles characterized by high-fat diets (HFDs) and low physical activity [1,9,10]. However, there is ongoing debate on this topic. Therefore, it is crucial to gain a better and more detailed understanding of the factors contributing to diabetic bone fragility and the mechanisms involved. This knowledge will help improve bone strength and reduce the risk of fragility fractures in individuals with T2DM. 

Advancements in technology and increased research efforts have significantly expanded our knowledge of the human microbiome. The human microbiome, which consists of approximately 100 trillion microorganisms primarily residing in the human gut, has become an increasingly important focus of scientific study. This surge in interest is driven by the realization that the microbiota contains a vast amount of genetic material, far exceeding the human genome, with over three million genes compared to the approximately 23,000 genes in the human genome [11]. Our growing understanding of the microbiota has revealed that they function as a hidden organ within the human body, producing numerous metabolites that may fulfill many host functions and influence the host’s phenotype and overall health [11,12]. Moreover, research has demonstrated that the human gut microbiota has profound effects on the immune system, influencing the development and progression of various metabolic diseases, including T2DM [12,13,14]. This finding is particularly significant considering that T2DM is one of the most prevalent metabolic diseases globally, with its incidence rapidly increasing. Additionally, there is emerging evidence suggesting that the microbiota play a role in regulating bone homeostasis in health and disease [15,16]. This implies that the microbiota may have a crucial role in the development of bone disorders such as osteoporosis. While studies have established the relationship between the gut microbiota and T2DM or bone metabolism separately, the exact role of the gut microbiota in the cause and mechanism of diabetic bone pathology remains unclear. Therefore, further research is needed in this area to gain a better understanding of the complex relationship between the gut microbiota, T2DM, and bone metabolism. 

Appropriate animal models of T2DM are necessary to address ethical concerns related to human research and to better understand the development and treatment of the disease [17,18]. One approach is to use animal models that closely resemble the human disease. While genetic models are expensive and do not accurately represent human T2DM, the most used animal model for T2DM involves feeding rodents an HFD and injecting them with a low dose of streptozotocin (STZ; approximately 35 mg/kg) [19]. HFDs can induce insulin resistance, hyperinsulinemia, visceral fat accumulation, and gut microbiota dysbiosis, but they do not lead to frank hyperglycemia or diabetes. This limits the ability to test interventions for controlling blood sugar levels and diabetes-related complications [20,21]. On the other hand, rodents can be induced to have hyperglycemia by injecting them with a relatively high dose of STZ (>50 mg/kg), which primarily destroys pancreatic beta cells and results in insulin deficiency, resembling type 1 diabetes rather than T2DM [22,23]. This model exhibits symptoms and characteristics like those of human type 1 diabetes and is not very responsive to interventions that target insulin resistance, making it less suitable for testing strategies aimed at improving insulin sensitivity [19,24]. Researchers have shown that combining an HFD with a low-dose STZ treatment can effectively create a rat model that mimics the natural progression and metabolic features of typical T2DM in humans. This model is cost-effective, easy to develop, and well suited for studying the underlying mechanisms of T2DM and evaluating potential therapeutic compounds for the treatment of T2DM and its associated complications [19]. 

Current clinical methods for diagnosing bone diseases mainly include physical evaluation and imaging technologies like X-rays, bone mineral density (BMD) tests, and CT/MRI scans [25,26,27]. However, these approaches may not fully grasp the unique aspects of osteopathogenesis in DM patients. This limitation stems from the complex nature of diabetic bone diseases and the lack of specific biomarkers. Moreover, although BMD tests serve as the benchmark for diagnosing osteoporosis, they might underestimate fracture risk in DM patients due to factors such as like altered bone geometry and poor bone quality. Furthermore, while imaging technologies provide valuable information about bone structure and integrity, they might not always detect subtle changes in bone quality or composition [26,27]. Given these constraints, there is a pressing need to devise novel diagnostic strategies to address the intricate processes of diabetic osteopathogenesis. 

This study aimed to evaluate the potential clinical usefulness of the gut microbiota in diabetic bone conditions by investigating the connections between the gut microbiota and lipid profiles or bone metabolism. 

## 2. Materials and Method

### 2.1. Experimental Animals

Male Sprague Dawley (SD) rats (6 weeks old) weighing 160–180 g were used. Body weight and food/water intake were monitored daily. The animals were housed in standard polypropylene cages with three rats per cage and were maintained under controlled room temperature (22 ± 2 °C) and humidity (55 ± 5%) with a 12:12 h light and dark cycle. Prior to the diet manipulation, all rats were provided with commercially available rat normal pellet diet (NPD) and water ad libitum. The Institutional Animal Care and Use Committee (IACUC) of Chang Gung Memorial Hospital approved the animal use protocol (permit no. 2018062502). All animal experiments were performed according to the guidelines for the Care and Use of Laboratory Animals as promulgated by the Institute of Laboratory Animal Resources, National Research Council, USA, and followed the Animal Protection Law by the Council of Agriculture, Executive Yuan, ROC. 

### 2.2. Study Design and Development of HFD-Fed and Low-Dose STZ-Treated T2DM Rats (Figure S1)

The rats (*n* = 16) were randomly divided into two dietary groups (*n* = 8/group) via Microsoft Office Home 2021—Excel and fed either an NPD or an HFD ad libitum for the first 5 weeks. The HFD consisted of 60% fat, 20% protein, and 20% carbohydrate, as a percentage of total kcal (Research Diets, Inc., New Brunswick, NJ, USA). The composition of the HFD is detailed in Table 1. After the initial 5 weeks of dietary manipulation, the HFD-fed rats (DM group) were injected with low-dose STZ (35 mg/kg) (Sigma-Aldrich, Merck KGaA, Darmstadt, Germany) intraperitoneally, while the NPD-fed rats (control group) were injected with vehicle citrate buffer [19]. Body weight (BW) and fasting plasma glucose (PGL) were regularly monitored. After 7 weeks of dietary manipulation, which was 2 weeks after the STZ or vehicle injection, hemoglobin A1c (HbA1c), non-fasting PGL, fasting PGL, fasting plasma insulin (PI), and blood ketones were measured. The homeostatic model assessment for insulin resistance (HOMA-IR), homeostatic model assessment of β-cell function (HOMA-β%), and disposition index (DI) were calculated according to the corresponding formula to determine the development of HFD-fed and low-dose STZ-treated T2DM rats [28]. The rats with a non-fasting PGL of ≥300 mg/dL and HbA1c ≥ 7% were considered diabetic and selected for further studies. Rats that did not meet these criteria were excluded from this study. The feed and water intake of the animals were also measured. The rats were allowed to continue to feed on their respective diets until the end of the study. A double-blind animal study was conducted: we established experimental and control groups, carried out interventions, and randomized categories. To minimize bias, data were collected blindly. Independent analysts, who were unaware of group assignments, ensured that the findings were objective and reliable. 

### 2.3. Blood Collection and Analytical Methods

Blood was collected from the lateral tail vein of rats, and the plasma was separated by means of centrifugation for 10 min at 3000 rpm. The plasma was then stored at −20 °C until PI determination was performed using the Mercodia Ultrasensitive Rat Insulin ELISA kit (Mercodia AB, Uppsala, Sweden). The serum concentration of procollagen type I amino-terminal propeptide (PINP) and cross-linked C-telopeptide of type I collagen (CTX-1) was determined using ELISA (IDS, Boldon, UK), following the manufacturer’s protocol. The serum levels of eotaxin, IP-10, leptin, LIX, fractalkine, and RANTES were measured using Luminex^®^ xMAP technology by Multiplex assay (Diasorin, Via Crescentino, Italy), according to the manufacturer’s instructions. Additionally, blood samples were tested for DM lipid profile, including TC, HDL-C, LDL-C, VLDL-C, and non-HDL-C. 

### 2.4. Micro-Computed Tomography Analysis

Bone samples were dissected, fixed in 10% formalin for 24 h, and then transferred to PBS at −20 °C pending analysis. A three-dimensional evaluation of bone mass and architecture in the metaphysis of the proximal femur and proximal tibia was performed using a µCT scanner (Skyscan 1076; Skyscan, Aartselaar, Belgium). For the femoral metaphysis, BMD and volumetric measurements were obtained from each right femur, starting from growth plate and extending 2 mm distally. Analyses of bone volume per total volume (BV/TV), bone surface per total volume (BS/TV), trabecular thickness (Tb. Th), trabecular number (Tb. N), trabecular separation (Tb. Sp), trabecular bone pattern factor (Tb. Pf), and structure model index (SMI) were conducted using TRI/3D-BON software (Version 6, RATOC System Engineering, Tokyo, Japan). Scans of the proximal femur were performed using the following parameters: a source voltage of 70 kV, a current of 141 mA, a 0.5 mm aluminum filter, and an isotropic voxel size of 11.55 µm. A BMD calibration of the µCT scanner was carried out daily for quality control with a phantom standard provided by the manufacturer. 

### 2.5. Compositional Analysis of the Gut Microbiota Using Pyrosequencing and Data Analysis 

Colon content homogenates in a PBS solution were immediately frozen at −80 °C and stored until further processing. Next-generation sequencing library preparations and Illumina MiSeq sequencing were conducted at GENEWIZ. Briefly, 30–50 ng DNA was used to generate amplicons using a MetaVx™ Library Preparation kit (GENEWIZ, Inc., South Plainfield, NJ, USA). The V3 and V4 hypervariable regions of prokaryotic 16S rDNA were selected for generating amplicons and following taxonomy analysis. DNA libraries were validated using an Agilent 2100 Bioanalyzer (Agilent Technology, Palo Alto, CA, USA), and quantified using a Qubit 2.0 Fluorometer (Invitrogen, Carlsbad, CA, USA). The QIIME data analysis package was used for 16S rRNA data analysis [29]. The effective sequences were grouped into operational taxonomic units (OTUs) using the clustering program VSEARCH (1.9.6) against the Silva 119 database pre-clustered at 97% sequence identity. Alpha diversity indices were calculated in QIIME from rarefied samples using the Chao1 index for richness, the Shannon index for evenness, and the Gini–Simpson index for dominance. Beta diversity was calculated using weighted and unweighted UniFrac [30]. Principal coordinate analysis (PCoA), non-metric multidimensional scaling (NMDS), and analysis of similarity (ANOSIM) were performed to evaluate differences between microbial communities [31]. An unweighted pair group method with arithmetic mean (UPGMA) tree from the beta diversity distance matrix was built [32]. The enriched and significant bacteria in each group were identified by means of linear discriminant analysis (LDA) combined with LDA effect-size (LEfSe) measurements [33]. LDA scores greater than 2.0 and 4.0 are considered significant and strongly significant by default, respectively [34]. To analyze the relationships of gut microbiota with environmental and biochemical factors, Spearman correlations were calculated pairwise, and a heatmap based on Spearman correlation coefficients was plotted [35]. A *r* value of the Spearman correlation coefficient represents the relevance of two groups, where *r* < 0 indicates negative correlation and *r* > 0 indicates positive correlation. *p* values were considered significant using a false discovery rate (FDR) of 25%. 

### 2.6. Statistical Analysis

Quantitative data are expressed as mean ± standard deviation (SD) unless otherwise indicated in the figure legends. Analysis was performed using Student’s *t*-test or analysis of variance (ANOVA). A *p* value of 0.05 was considered significant and is shown as *p* < 0.05 (*), *p* < 0.01 (**), or *p* < 0.001 (***). 

## 3. Results

### 3.1. HFD-Fed and Low-Dose STZ-Induced T2DM Rats Have Decreased Bone Density, Reduced Bone Turnover, and Altered Gut Microbiota

**Figure 1:**
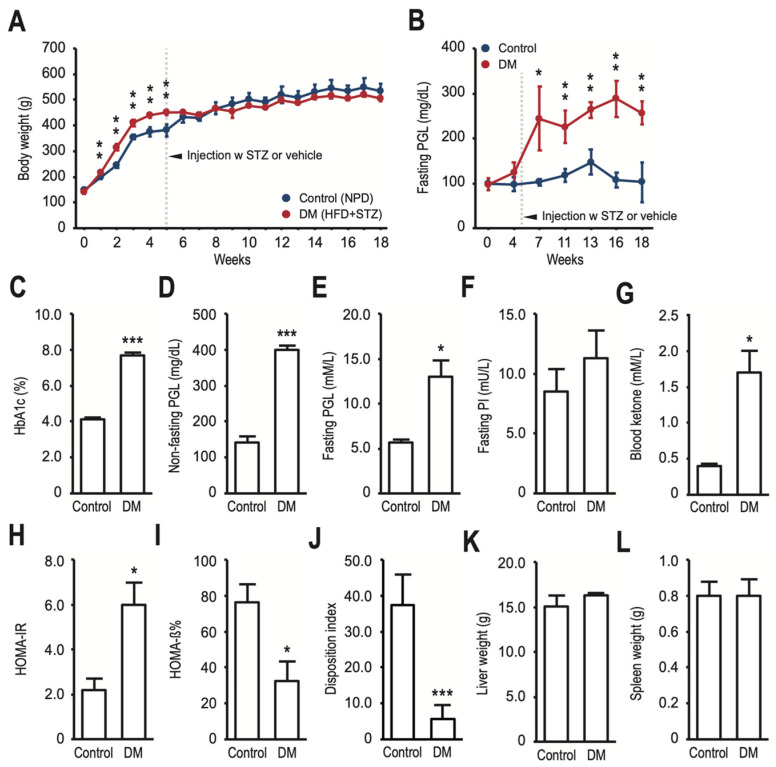
Creating a rat model mimicking T2DM in humans through a combination of HFD and low-dose STZ treatment. Male SD rats (6 weeks old) were randomly assigned to either NPD or HFD for 18 weeks at the start of the experiment. After 5 weeks of dietary manipulation, the experimental rats (DM group) received intraperitoneal injections of low-dose STZ, while the control rats were injected with vehicle citrate buffer. The rats’ body weight (**A**) and fasting plasma glucose (PGL) (**B**) were regularly monitored. At week 7, the researchers measured HbA1c (**C**), non-fasting PGL (**D**), fasting PGL (**E**), fasting plasma insulin (PI) (**F**), and blood ketones (**G**) to determine the development of HFD-fed and STZ-treated T2DM rats, and HOMA-IR (**H**), HOMA-β% (**I**), and disposition index (**J**) were calculated using the corresponding formula. At the end of the experiment (week 18), the rats were sacrificed, and their liver weights (**K**) and spleen weights (**L**) were determined. The data are expressed as means ± SD (error bars) of eight rats per group. Statistical significance was assessed with * *p* < 0.05, ** *p* < 0.01, and *** *p* < 0.001 vs. control at the same time point.

To create T2DM models in normal SD rats, a combination of HFD and low-dose STZ treatment was used. Table 2 shows that feeding HFD for 5 weeks resulted in a significant increase in body weight, fasting PI, HOMA-IR, leptin, RANTES, and fractalkine levels in rats compared to NPD-fed rats. However, fasting PGL levels did not show a significant increase. The STZ injection at 35 mg/kg on week 5 of HFD manipulation resulted in a new-onset increase in fasting PGL and IP-10 levels and a further increase in HOMA-IR and fractalkine levels 2 weeks later in rats compared to HFD-fed only rats (Table 2). The positive effect of HFD on body weight was abolished by the low-dose STZ administration (Figure 1A), which significantly increased the level of fasting PGL throughout the remaining time of the study (Figure 1B). On the 7th week of dietary manipulation (i.e., 2 weeks after the vehicle or STZ injection), the HFD/STZ-treated rats had a significant increase in HbA1c (7.7 ± 0.1% vs. 4.1 ± 0.1%, *p* < 0.001) (Figure 1C), non-fasting PGL (398.4 ± 6.4 mg/dL vs. 141.0 ± 9.0 mg/dL, *p* < 0.001) (Figure 1D), and fasting PGL (13.00 ± 1.03 mM/L vs. 5.68 ± 0.12 mM/L, *p* < 0.05) (Figure 1E) levels, despite a non-significant increase in fasting PI level (11.30 ± 1.30 mU/L vs. 8.51 ± 1.02 mU/L, *p* = 0.248) (Figure 1F) compared to NPD-fed rats. In this study, HFD/STZ-treated rats met the criteria for diabetes mellitus (i.e., a non-fasting PGL of ≥300 mg/dL and HbA1c ≥ 7%). The NPD-fed only rats were designated as the control group. Compared with those in the control group, rats in the DM group had significantly higher blood ketone levels (1.7 ± 0.2 mM/L vs. 0.4 ± 0.0 mM/L, *p* < 0.05) (Figure 1G), indicating the inability of the β-cells to produce sufficient insulin in a condition of hyperglycemia, which characterizes the transition from insulin resistance to T2DM [36]. HOMA-IR (5.96 ± 0.57 vs. 2.18 ± 0.30, *p* < 0.05) (Figure 1H), HOMA-β% (32.54 ± 17.23 vs. 76.4 ± 9.92, *p* < 0.05) (Figure 1I), and disposition index (5.55 ± 2.21 vs. 37.37 ± 4.91, *p* < 0.001) (Figure 1J) were then calculated according to the corresponding formula to confirm the establishment of the T2DM model in normal SD rats. At the end of the study (week 18), rats were sacrificed, and their liver weights (Figure 1K) and spleen weights (Figure 1L) were determined. There were no significant differences between the two groups. 

**Figure 2:**
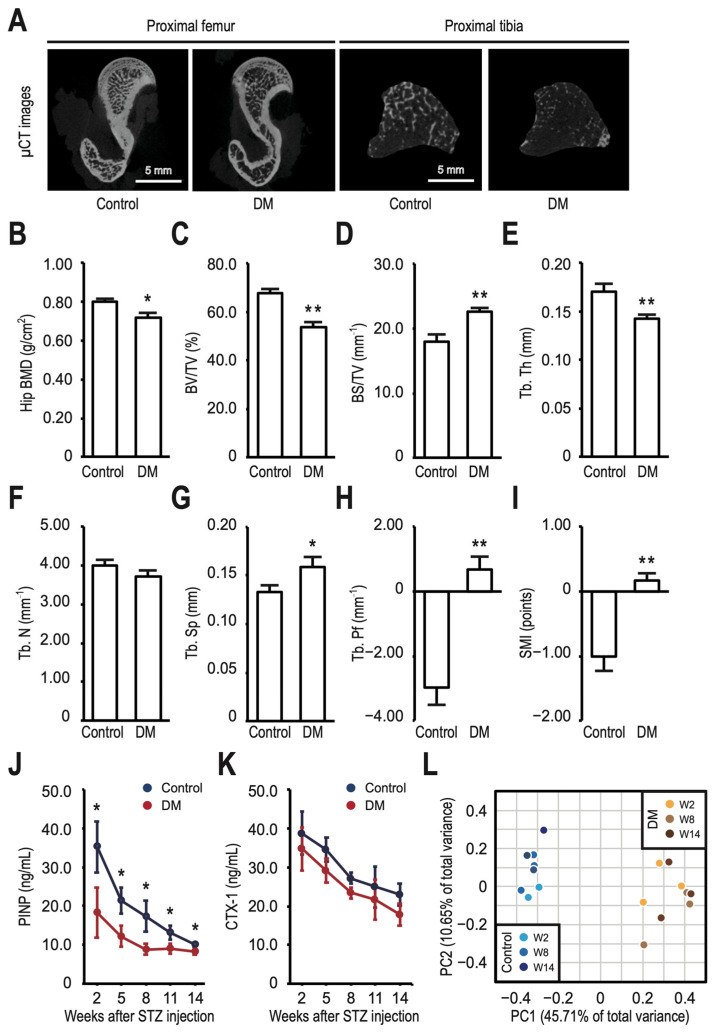
T2DM rats exhibit decreased bone density, reduced bone turnover, and altered gut microbiota. (**A**) Micro-computed tomography (µCT) images were taken of the proximal femur and proximal tibia at week 18 for the control and DM groups. The following bone parameters were analyzed for the proximal femur specimens using µCT: hip bone mineral density (BMD) (**B**), bone volume per total volume (BV/TV) (**C**), bone surface per total volume (BS/TV) (**D**), trabecular thickness (Tb. Th) (**E**), trabecular number (Tb. N) (**F**), and trabecular separation (Tb. Sp) (**G**). Trabecular bone pattern factor (Tb. Pf) (**H**) and structure model index (SMI) (**I**) were calculated to determine the connectivity and geometry of the trabecular bone. Procollagen type I amino-terminal propeptide (PINP) (**J**) and cross-linked C-telopeptide of type I collagen (CTX-1) (**K**) were monitored every 3 weeks following STZ injection. (**L**) Gut microbial structure was analyzed using principal coordinates analysis (PCoA) based on the Bray–Curtis distance matrix. PC1 and PC2 represent principal coordinate 1 and principal coordinate 2, respectively. W2, W8, and W14 stand for week 2, 8, and 14, respectively. All data are presented as means ± SD (error bars) of eight rats per group. Statistically significant differences were observed between the DM group and the control group at the same time point: * *p* < 0.05 and ** *p* < 0.01 vs. control.

Compared to the control group, rats in the DM group showed significantly decreased bone mass (Figure 2A), indicated by a decreased hip BMD (0.716 ± 0.019 g/cm^2^ vs. 0.801 ± 0.006 g/cm^2^, *p* < 0.05) (the BMD in the trabecular or cortical bone of the distal femur was within the normal range, Figure S2), BV/TV (53.5 ± 1.3 vs. 67.7 ± 0.9%, *p* < 0.01), Tb. Th (0.142 ± 0.002 vs. 0.170 ± 0.004 mm, *p* < 0.01), and Tb. N (3.7 ± 0.1 vs. 4.0 ± 0.1 mm^−1^, *p* = 0.10). Furthermore, BS/TV (22.6 ± 0.3 vs. 17.9 ± 0.6%, *p* < 0.01), Tb. Sp (0.159 ± 0.005 vs. 0.133 ± 0.004 mm, *p* < 0.05), Tb. Pf (0.7 ± 0.2 vs. −3.0 ± 0.3 mm^−1^, *p* < 0.01), and SMI (0.17 ± 0.05 vs. −1.00 ± 0.13, *p* < 0.01) were all significantly increased (Figure 2B–I). The serial measurement of PINP and CTX-1 levels, as bone turnover markers at five time points (week 2, 5,8,11, and 14 after STZ injection), revealed that rats in the DM group had reduced bone turnover compared to those in the control group (Figure 2J,K), although the difference in CTX-1 level between groups did not reach statistical significance. Meanwhile, we also examined the effect of HFD/STZ treatment on gut microbial diversity. The PCoA scaling plot, based on the Bray–Curtis distance matrix of dissimilarity, revealed that samples in each group clearly clustered together, indicating a lower intragroup variability of the gut microbiota than average. However, there was a substantial intergroup difference in the overall structure of the gut microbiota (Figure 2L).

### 3.2. There Were Significant Differences in the Taxonomic Profile of Gut Microbiota between the Control and T2DM Rats

**Figure 3:**
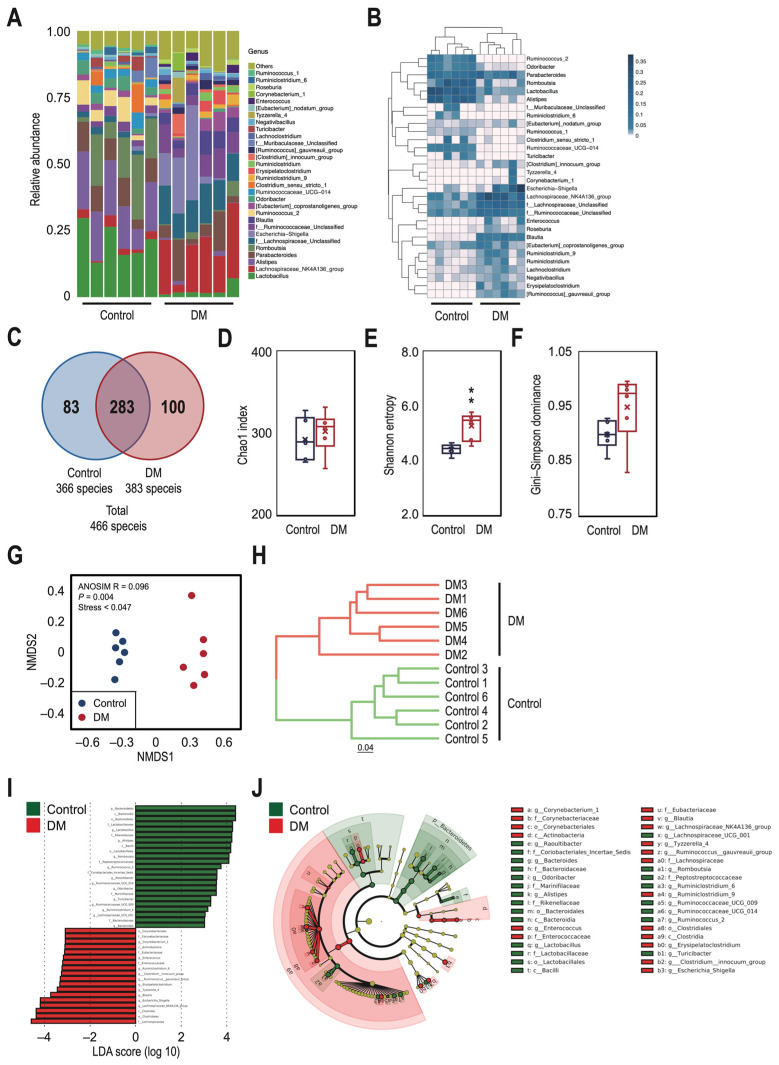
Marked differences in the taxonomic profile of gut microbiota were observed between the control and T2DM rats. (**A**) Composition of microbiota at the genus level of the control and DM groups. (**B**) A heatmap displaying the abundance information of the top 30 OTUs with the highest abundance, as well as the similarity and difference across OTUs and samples by means of similarity clustering. (**C**) A Venn diagram showing the number of shared taxa at the genus level in the control and DM groups. Alpha diversity, measured by the Chao1 index (**D**), Shannon entropy (**E**), and Gini–Simpson dominance (**F**), was assessed to determine the community richness, evenness, and dominance of the gut microbial structure in the control and DM groups. (**G**) Non-metric multidimensional scaling (NMDS) ordination of gut microbial communities within control (blue) and DM (red) groups. Symbols represent samples, and distances between samples represent similarities between samples. The significance value refers to the analysis of similarity (ANOSIM) test for differences in community composition between groups. (**H**) A multiple-sample similarity phylogenetic tree was built using the unweighted pair group method with arithmetic mean (UPGMA) clustering method. Each branch in the figure represents a sample. Green and red represent the control group and the DM group, respectively. (**I**) A histogram of the linear discriminant analysis (LDA) scores presents species whose abundance showed significant differences between the control and DM groups. The length of each bin, namely, the LDA score, represents the effect size. (**J**) A LEfSe (LDA effect size) cladogram was constructed to indicate the evolutionary relationships of different species. The nodes in red represent taxa enriched in the DM group, and those in green represent taxa enriched in the control group. Data are expressed as medians ± IQR (box span) of six rats per group. Statistical significance was assessed with ** *p* < 0.01 vs. control.

To determine the taxonomic shift pattern in the T2DM rat model, bacterial taxonomic profiling was analyzed at the genus level (Figure 3A and Figure S3). The relative abundance of genus-level OTUs in samples from the control and T2DM rats was significantly different, with the T2DM rats having a higher proportion of enriched sequences than the control group (Figure 3B and Figure S4). Although 283 OTUs were common between the two groups, the total number of OTUs in the control and T2DM rats was 366 and 383, respectively (Figure 3C). The Chao1 and Gini–Simpson dominance indices did not differ significantly (*p* = 0.54 and 0.10, respectively), but the Shannon entropy index of the T2DM group was significantly higher than that of the control group (*p* < 0.01) (Figure 3D–F and Figure S5). Beta diversity showed significant differences between group compositions (ANOSIM R = 0.096, *p* = 0.004, Stress < 0.047) (Figure 3G), and the UPGMA clustering based on the Bray–Curtis distance matrices also confirms significant dissimilarity of the microbial communities between the control and T2DM rats (Figure 3H). A further analysis of the gut microbiota was conducted using LDA effect size to detect significant differences at the genus level between the two groups (Figure 3I and Figure S6) and classify the bacteria at the corresponding classification level on a cladogram (Figure 3J and Figure S7). The results showed that *Lactobacillus*, *Alistipes*, and *Romboutsia* were more abundant in the control group (LDA ≥ 4.0), while the *Lachnospiraceae NK4A136* and *Escherichia Shigella* were more abundant in the T2DM group (LDA ≤ −4.0). Additionally, the majority of the Bacteroidetes phylum was more abundant in the control group (LDA > 4.0).

### 3.3. T2DM Rats Have a Higher Ratio of Firmicutes to Bacteroidetes in Their Gut Microbiota, Which Correlates with Decreased Bone Density and Reduced Bone Turnover

**Figure 4:**
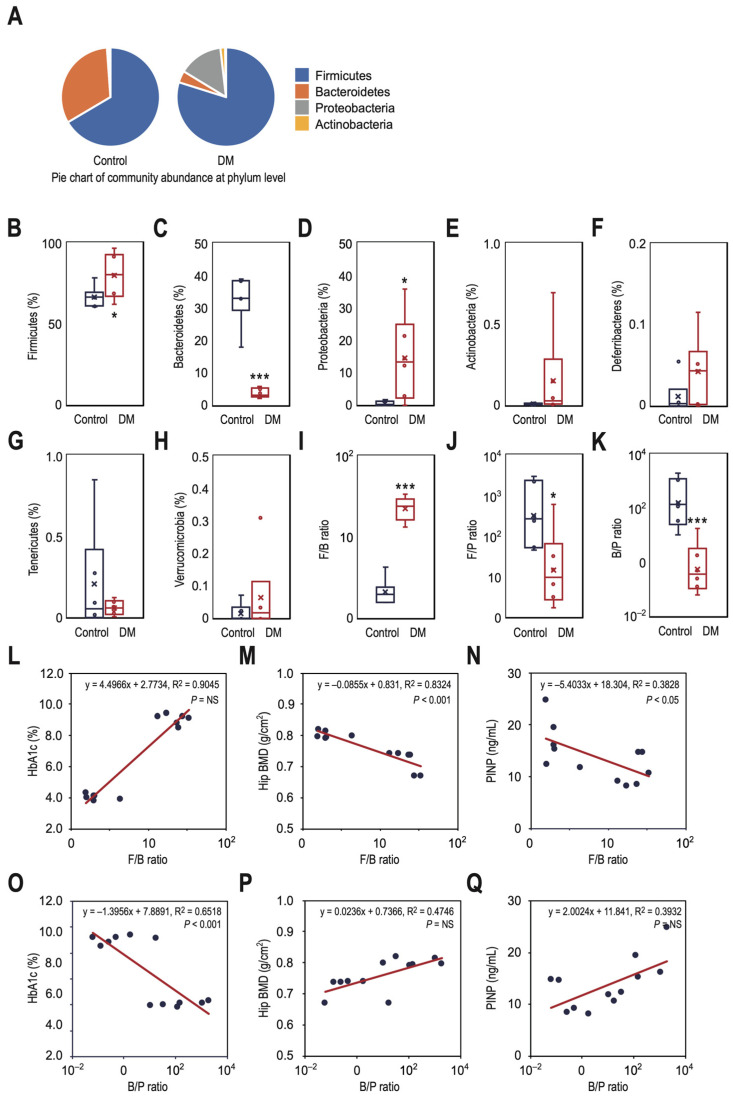
The gut microbiome of T2DM rats shows a higher ratio of Firmicutes to Bacteroidetes, which is associated with lower bone density and decreased bone turnover. (**A**) The community abundance at the phylum level of the control and DM groups is shown. The differences in proportion and patterns of relative abundance of the seven identified phyla, including Firmicutes (**B**), Bacteroidetes (**C**), Proteobacteria (**D**), Actinobacteria (**E**), Deferribacteres (**F**), Tenericutes (**G**), and Verrucomicrobia (**H**), were evaluated for the detection of a major shift in the microbial composition. The F/B (**I**), F/P (**J**), and B/P (**K**) ratios were measured to investigate the microbial composition differences. The correlations of HbA1c, hip BMD, and PINP against the F/B ratio (**L**–**N**) and B/P ratio (**O**–**Q**) on a logarithmic scale with a base of 10 were also plotted to explore whether these ratios could be used as diagnostic makers. Data are expressed as medians ± IQR (box span) of six rats per group. * *p* < 0.05 and *** *p* < 0.001 vs. control.

To compare the DM group with the control group in global phylum-level diversity of gut microbiota, we analyzed the phylogenetic composition at the phylum level, as shown in Figure 4A. DM rats had a higher relative abundance of Firmicutes (79.8% vs. 66.5%, *p* < 0.05) and Proteobacteria (14.5% vs. 0.6%, *p* < 0.05), but a lower relative abundance of Bacteroidetes (3.8% vs. 32.4%, *p* < 0.001) in their gut compared to control rats (Figure 4B–D). We did not find any intergroup differences in the relative abundance of Actinobacteria (Figure 4E), Deferribacteres (Figure 4F), Tenericutes (Figure 4G), and Verrucomicrobia (Figure 4H). When we transformed the data by rate function in log format (base 10), DM rats had a significantly higher level of the F/B ratio (1.35 vs. 0.32, *p* < 0.001), but a lower level of the F/P (1.17 vs. 2.51, *p* < 0.05) and B/P ratios (−0.18 vs. 2.19, *p* < 0.001) than control rats (Figure 4I–K). To determine whether these ratios could be used as potential diagnostic markers, we calculated the correlations of HbA1c, hip BMD, and PINP against the F/B (Figure 4L–N) and B/P ratios (Figure 4O–Q) on a log scale with a base of 10. The F/B ratio had a significant negative correlation with hip BMD (R^2^ = 0.8324, *p* < 0.001) and PINP level (R^2^ = 0.3828, *p* < 0.05), but a non-significant positive correlation with HbA1c level (R^2^ = 0.9045, *p* = NS). On the other hand, the B/P ratio had a significant negative correlation with HbA1c level (R^2^ = 0.6518, *p* < 0.001), but a non-significant positive correlation with hip BMD (R^2^ = 0.4746, *p* = NS) and PINP level (R^2^ = 0.3932, *p* = NS). 

### 3.4. Changes in the Relative Abundance of Predominant Genera Correlate with Decreased Bone Density and Reduced Bone Turnover in T2DM Rats

**Figure 5:**
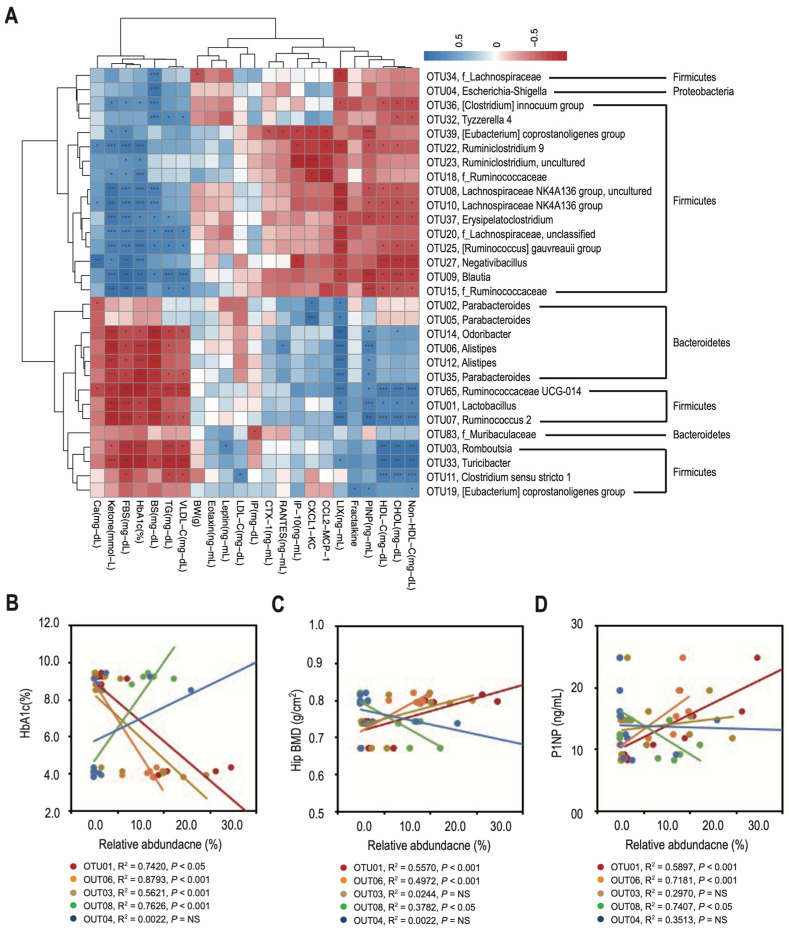
Correlation between changes in predominant genera and decreased bone density and reduced bone turnover in T2DM rats. (**A**) The heatmap shows correlation (based on Spearman analysis) between environmental/biochemical factors and OTUs. The column labels at the bottom indicate the environmental or biochemical factors, while the labels on the right indicate the OTUs. Shades of blue or red represent positive or negative correlations, respectively. Dendrograms display different clustering among the various environmental/biochemical factors and OTUs. (**B**–**D**) We measured and plotted the correlations of HbA1c, hip BMD, and PINP against the relative abundance of predominant genera to determine if these microbial distributions could be used as diagnostic makers or therapeutic targets. The data are expressed as means ± SD (error bars) of six rats per group. * *p* < 0.05, ** *p* < 0.01, and *** *p* < 0.001 vs. control.

To identify the differences associated with phenotyping in T2DM rats, we examined the correlations between various environmental and biochemical factors and the composition of the top 30 OTUs with the highest abundance. Using clustering heatmaps, we observed two groups of bacterial taxa with fundamentally contrasting relationships. Bacteroidetes (e.g., *Alistipes*) and some Firmicutes (e.g., *Lactobacillus*) were strongly correlated with PINP levels, and negatively correlated with DM lipid profiles (e.g., HbA1c, blood sugar, TG, and VLDL levels). In contrast, other Firmicutes (e.g., *Lachnospiraceae*) were positively correlated with DM lipid profiles but negatively correlated with PINP levels (Figure 5A). There was no significant correlation between CTX-1 levels and both groups of bacterial taxa. To determine whether the predominant genera (LDA ≥ 4.0) shown in Figure 3I could be used as potential diagnostic markers and/or therapeutic targets, we calculated the correlations of HbA1c (Figure 5B), hip BMD (Figure 5C), and PINP (Figure 5D) against the relative abundance of *Lactobacillus* (OTU01), *Alistipes* (OTU06), *Romboutsia* (OTU03), *Lachnospiraceae NK4A136 group* (OTU08), and *Escherichia Shigella* (OTU04). HbA1c levels negatively correlated with the relative abundances of *Lactobacillus* (R^2^ = 0.7420, *p* < 0.05), *Alistipes* (R^2^ = 0.8793, *p* < 0.001), and *Romboutsia* (R^2^ = 0.5621, *p* < 0.001), but positively correlated with that of the *Lachnospiraceae NK4A136 group* (R^2^ = 0.7626, *p* < 0.001). There was no correlation between HbA1c levels and the relative abundance of *Escherichia Shigella* (R^2^ = 0.0022, *p* = NS). In contrast, hip BMD and PINP levels positively correlated with the relative abundances of *Lactobacillus* (R^2^ = 0.5570 and 0.5897, respectively, both *p* < 0.001) and *Alistipes* (R^2^ = 0.4972 and 0.7181, respectively, both *p* < 0.001), but negatively correlated with that of the *Lachnospiraceae NK4A136 group* (R^2^ = 0.3782 and 0.7404, respectively, both *p* < 0.05). There was no correlation between hip BMD/PINP levels and the relative abundance of *Romboutsia*/*Escherichia Shigella* (R^2^ = 0.0244/0.2970 and 0.0022/0.3513, respectively, all *p* = NS).

## 4. Discussion

This study presents a valuable tool for exploring the link between T2DM and osteoporosis, highlighting the potential role of gut microbiota in this relationship. The systematic experiments revealed the following: (i) A non-obese T2DM model can be induced in normal SD rats without genetic manipulation by combining an HFD and low-dose STZ treatment. This model closely resembles the characteristics of most diabetic patients among Asian races [19,37]. (ii) T2DM rats show lower bone mass and reduced bone turnover markers compared to control rats. (iii) There is a notable difference in gut microbiota between T2DM rats and control rats [13]. T2DM rats have a higher relative abundance of Firmicutes and Proteobacteria, but a lower relative abundance of Bacteroidetes at the phylum level. The F/B ratio and the B/P ratio demonstrate negative correlations with hip BMD and PINP levels, and HbA1c levels, respectively. (iv) At the genus level, the control group has a higher abundance of *Lactobacillus*, *Alistipes*, and *Romboutsia*, while T2DM rats have a higher abundance of *Lachnospiraceae NK4A136* and *Escherichia Shigella*. Additionally, the relative abundance of *Lactobacillus* and *Alistipes* shows a significantly negative correlation with HbA1c levels, but a positive correlation with hip BMD and PINP levels. On the other hand, the relative abundance of *Lachnospiraceae* exhibits the opposite correlation. These findings have important implications for the development of new theragnostic strategies for diabetic bone diseases. 

T2DM and its associated complications arise from insulin resistance and β-cell dysfunction, making it a complex condition. The disease progresses through two stages: compensation and decompensation. During the compensation stage, the body compensates for insulin resistance by increasing insulin secretion to maintain euglycemia. However, as the disease advances, the body becomes unable to produce enough insulin to meet the demand, leading to hyperglycemia, frank diabetes, and ketosis [38]. While there are several animal models available for studying T2DM, only a few can accurately replicate the natural progression of the disease or the metabolic characteristics observed in humans [17,19,39,40]. This study demonstrates that the currently used animal model can replicate both the compensation and decompensation stages of human T2DM. Prior to administering a low-dose STZ injection, rats fed an HFD showed a significant increase in body weight, fasting PI, and HOMA-IR, but did not exhibit elevated fasting PGL levels. These characteristics are similar to those observed during the compensation stage of T2DM in humans. However, administering a low dose of STZ resulted in a decompensated disease pattern in HFD-fed rats with insulin resistance. This led to frank hyperglycemia and ketosis, despite the rats having fasting PI levels comparable to those in NPD-fed rats. Interestingly, the positive effect of an HFD on body weight was counteracted by the low-dose administration of STZ, resulting in characteristics similar to those of non-obese T2DM, which is commonly observed in diabetic patients of Asian descent [37]. Based on these observations, the current animal model represents a progressive metabolic condition that closely mirrors the natural progression of individuals at risk of developing T2DM. Furthermore, subjects at the decompensated T2DM stage exhibited osteoporotic bone pathology and a simultaneous change in gut microbiota ecology. 

Our μCT results indicate that T2DM rats have lower bone mass compared to control rats. This is characterized by BV/TV fraction, Tb. Th, Tb. N, along with increased BS/TV, Tb. Sp, Tb. Pf, and SMI. Additionally, bone turnover markers, specifically PINP and CTX-1, were significantly inhibited in T2DM rats compared to normal rats. These findings suggest that T2DM may lead to a decrease in bone formation relative to bone resorption, resulting in compromised bone microarchitecture, mineralization, and strength. Recent studies have also shown a higher risk of bone fragility and post-fracture mortality in patients with T2DM compared to the general population [7,41,42]. Combining these studies with our findings, it can be inferred that T2DM is associated with bone-related complications. However, this poses a significant challenge as the development of bone fragility caused by T2DM could further impact quality of life and increase healthcare costs [43,44]. Currently, no specific drug has been identified for the direct treatment of diabetic bone disease [8,25]. This may be due to the lack of an appropriate assessment method to accurately establish the relationship between bone status and DM, as well as the absence of a suitable marker for such evaluations [25,26]. Recently, assessing BMD and trabecular bone score (TBS) has been considered as a promising clinical strategy for detecting diabetic bone problems [25,26,27]. However, evidence has shown that BMD alone may not adequately reflect changes in bone microarchitecture. Furthermore, while BMD levels decrease in T1DM, they may remain normal or even increase in T2DM. Additionally, TBS evaluation may be more suitable for detecting the early-onset bone defects in DM [25,26]. Therefore, it appears that these current methods or strategies still have conflicting issues that require further investigation. In our BMD evaluation, we observed a reduced level of hip BMD in T2DM rats compared to control rats, seemingly contradicting clinical observations. However, our data also revealed that the BMD levels in the trabecular or cortical bone of the distal femur were similar between control and T2DM rats. Therefore, we propose that this puzzling difference may likely stem from limitations inherent to the animal model used in our study.

The mechanistic study of diabetic bone disease suggests that several factors may contribute to bone fragility in individuals with T2DM. These factors include the formation and aggregation of advanced glycated matrix proteins, such as glycated collagen I, which could degrade bone mineralization and stabilization. Additionally, an inflammatory and/or oxidative stress microenvironment, insulin resistance progression, alterations in lipid profiles, and a hyperglycemic/hypercholesterolemic environment may also play a role [5,25]. In these scenarios, signaling factors from pathways like the RAGE (receptor for advanced glycation end products)-JAK pathway, NF-κB pathway, and Wnt/β-catenin pathway have been shown to regulate bone homeostasis, making them potential markers for assessing diabetic osteopathogenesis [5,25]. Furthermore, recent evidence has revealed interesting changes in gut microbiota during the progression of DM or bone disease [45,46]. Therefore, investigating the specific gut bacteria that influence the development of diabetic bone disease could be a new approach for developing theragnostic tools or drugs. Additionally, combining BMD/TBS detection methods with the study of mechanistic molecules and/or gut microbiota alterations may offer a promising strategy for evaluating the status of diabetic osteopathogenesis and could have potential benefits for future therapeutic interventions. 

Our research has uncovered a complex relationship between the gut microbiota, T2DM, and bone health. Our findings indicate that T2DM rats have a higher abundance of communities and species richness/evenness compared to normal rats. Specifically, at the phylum level, we observed elevated levels of Firmicutes and Proteobacteria in T2DM rats, while normal rats showed higher levels of Bacteroidetes. Further analysis revealed the following characteristics in T2DM rats: (i) a higher F/B ratio, which indicates an increased risk of osteoporosis, as supported by a significant negative correlation between the F/B ratio and hip BMD and PINP levels; and (ii) a lower B/P ratio, indicating an increased risk of DM, as evidenced by a significant negative correlation between the B/P ratio and HbA1c levels. The predominant phyla (>90%) of gut bacteria include Firmicutes, Bacteroidetes, Proteobacteria, Actinobacteria, Fusobacteria, Tenericutes, and Verrumicrobia [47,48]. The correlation of these phyla with disease development has been further examined based on individual abundance and ratio modes. However, while Firmicutes and Bacteroidetes have been extensively studied and shown to be associated with inflammation, obesity, DM, and osteoporosis through the measurement of F/B or B/F ratio, it is important to note that the F/B or B/F ratio cannot universally serve as an assessment index for these diseases due to conflicting results in different studies [45,48,49,50]. This discrepancy may be attributed to various factors that affect gut microbiota, such as diet, physical activity, age, gender, and even analytic methods [45]. Our results suggest that two bacteria ratios, F/B and B/P, may be associated with bone fragility and DM development, respectively. While the abundance of Firmicutes and Bacteroidetes and the F/B ratio in DM and osteoporosis align with the majority of studies, the research on the abundance of Proteobacteria in the development of DM and bone diseases, from both other studies and our own, remains inconclusive [45,49,51]. 

At the genus level, higher levels of *Lactobacillus*, *Alistipes*, and *Romboutsia*, and lower levels of *Lachnospiraceae NK4A136* and *Escherichia Shigella* were observed in the control and T2DM rats, respectively. Further analysis suggested that *Lactobacillus* and *Alistipes* have a negative correlation with HbA1c levels but a positive correlation with bone turnover levels, while *Lachnospiraceae NK4A136* shows the opposite correlation. Considering the concept of probiotics, specific bacteria genera/species identified in the analysis of gut microbiota will be further explored for their potential application in clinical treatment and dietary supplementation. *Lactobacillus*, *Alistipes*, and *Lachnospiraceae NK4A136* have been identified as potential indicators of microbiota dysbiosis, indicating their significant correlation with health and disease development [52,53,54,55,56]. Certain bacteria in the *Lactobacillus* genus have been found to have varied benefits in regulating glucose and lipid levels, reducing inflammation, and preventing bone loss by modifying the gut microbiota with increased probiotic bacteria and decreased harmful bacteria or by influencing the immune system [52,53]. *Alistipes* is a newly discovered bacterium in the Bacteroidetes phylum that has shown a multifunctional role in regulating the development of DM, bone diseases, and cancers [54,55]. While there is still debate, it is considered a promising candidate as a probiotic. Similarly, *Lachnospiraceae NK4A136* has shown a positive correlation with the development of DM, but some studies suggest its probiotic role in attenuating inflammation [56]. Taking all these statements into account, our findings are supported by previous studies, but further investigation into the clinical and dietary applications of these gut bacteria is still necessary. 

T2DM is recognized as a chronic inflammatory disease, with an unhealthy diet being one of its causative factors. Hyperglycemia is its major symptom and is also considered to be the initiator of the inflammatory microenvironment and subsequent complications. Furthermore, these processes have been further correlated with alterations in gut microbiota composition. As a result, accumulating evidence suggests that nutritional interventions may be a potential method of controlling blood sugar levels and mitigating complications by re-regulating the gut microbiota [57,58]. The gut–immune–bone axis is considered a significant link connecting changes in the microbiota to the development and treatment of bone diseases, particularly in the context of aging. The concept of this axis suggests that dietary bioactives (such as dietary lipids, polysaccharides, polyphenol, vitamins, and metals) may support and maintain the balance of probiotics, and subsequently, their metabolites, such as short-chain fatty acids and bile acids, could further regulate the immune system [59]. This immunomodulatory property could potentially ameliorate the development of bone diseases, such as osteoarthritis (by inhibiting chondrocyte inflammation) and osteoporosis (by remodeling/rebalancing osteoblast and osteoclast activity) [59,60,61,62]. Moreover, the gut microbiota plays a critical role in regulating intestinal calcium absorption and vitamin D metabolism, essential for mitochondrial quality control (metabolic homeostasis), macrophage polarization (immunomodulation), and bone cell mineralization [59,61,62,63]. Additionally, Omega-3 fatty acids, abundant in fish oil, have shown promise in modulating immune responses and inhibiting osteoclast activity, offering protective effects against bone resorption in T2DM patients, especially in older adults [57,58,59]. Considering these reports, it has been proposed that dietary supplementation with beneficial foods for T2DM patients could not only control blood sugar levels but also improve the gut microbiota composition, thereby modulating immune responses and ultimately affecting the development of complications, including diabetic osteopathogenesis.

## 5. Conclusions

The gut microbiota are a complex entity in the human body that influences various physiological and pathophysiological processes. It does so by producing metabolites and regulating the human immune system and signal pathways. Numerous studies have investigated the connection between changes in the gut microbiota and the development of diseases. However, the specific role and definition of certain bacteria phyla and genus abundance, as well as their ratios, remain unclear and require further investigation. This study reveals that alterations in the gut microbiota may contribute to bone fragility in individuals with T2DM. We propose that the F/B and B/P ratios could be correlated with diabetic osteopathogenesis. Nevertheless, additional research and analysis, incorporating animal models and human samples with varying parameters, are necessary to fully evaluate the potential theragnostic applications of our findings in the context of diabetic osteopathogenesis.

## Figures and Tables

**Table 1:** Composition of HFD (D12492i). Total: 733.85 g.

Class Description	Ingredient	Grams
Protein	Casein, Lactic, 30 Mesh	200.00 g
Protein	Cystine, L	3.00 g
Carbohydrate	Lodex 10	125.00 g
Carbohydrate	Sucrose, Fine Granulated	72.80 g
Fiber	Solka Floc, FCC200	50.00 g
Fat	Lard	245.00 g
Fat	Soybean Oil, USP	25.00 g
Mineral	S10026B	50.00 g
Vitamin	Choline Bitartate	2.00 g
Vitamin	V10001C	1.00 g
Dye	Dye, Blue FD&C #1, Alum. Lake 35–42%	0.05 g

**Table 2:** Effects of HFD and HFD + STZ on body weight, biochemical parameters, and levels of cytokines in rats.

Parameters	NPD (*n* = 8)	HFD (*n* = 8)	HFD + STZ (*n* = 8)
Body weight (g)	374.6 ± 19.8	429.3 ± 8.8 **	419.5 ± 9.6 *
Fasting PGL (mM/L)	5.44 ± 0.48	5.84 ± 0.47	13.00 ± 1.78 *^, ##^
Fasting PI (mU/L)	3.45 ± 0.72	7.80 ± 0.83 *	11.30 ± 2.26 *
HOMA-IR	0.86 ± 0.24	2.16 ± 0.38 *	5.96 ± 0.99 **^, ##^
Leptin (ng/mL)	4687.0 ± 902.6	13339.5 ± 1723.9 **	13073.0 ± 3112.0 *
RANTES (CCL5) (ng/mL)	6830.0 ± 787.8	10413.0 ± 1379.1 *	13043.0 ± 1420.4 *
Eotaxin (CCL) (ng/mL)	26.5 ± 4.3	25.4 ± 3.0	36.3 ± 4.2
IP-10 (CXCL10) (ng/mL)	219.6 ± 23.7	271.9 ± 23.8	365.6 ± 37.1 *^, #^
Fractalkine (CX3CL1) (ng/mL)	33.6 ± 1.3	60.7 ± 8.5 *	36.7 ± 4.8 ^#^
LIX (CXCL5) (ng/mL)	4489.0 ± 461.7	5822.6 ± 503.6	4747.1 ± 348.8

Data are presented as mean ± SEM. * *p* < 0.05 vs. NPD group. ** *p* < 0.01 vs. NPD group. ^#^, *p* < 0.05 vs. HFD group. ^##^, *p* < 0.01 vs. HFD group. The abbreviations denote PGL: plasma glucose; PI: plasma insulin; and HOMA-IR: Homeostasis Model Assessment-Insulin Resistance.

## Data Availability

The data that support the findings of current study are available from the corresponding author upon reasonable request.

## Supplementary Materials

The following supporting information can be downloaded at: https://www.mdpi.com/article/10.3390/nu16081220/s1, Figure S1: Study design scheme; Figure S2: uCT parameters derived from the trabecular or cortical bone of the distal femur; Figure S3: Composition of microbiota at the genus level of the control and DM groups; Figure S4: A heatmap displaying the abundance information of the top 30 OTUs with the highest abundance, as well as the similarity and difference across OTUs and samples by means of similarity clustering; Figure S5: Comparison of gut microbiota diversity in control and T2DM rats; Figure S6: A histogram of the linear discriminant analysis (LDA) scores presents species whose abundance showed significant differences between the control and DM groups); Figure S7: A LEfSe (LDA effect size) cladogram was constructed to indicate the evolutionary relationships of different species.
