# Supplementary material for: Diabetic Rats Induced Using a High-Fat Diet and Low-Dose Streptozotocin Treatment Exhibit Gut Microbiota Dysbiosis and Osteoporotic Bone Pathologies
Source: Nutrients. 2024 Apr 19;16(8):1220. doi: 10.3390/nu16081220 (PMC11054352; doi:10.3390/nu16081220)
Supplement: Supplementary file 1 [file nutrients-16-01220-s001.zip › nutrients-2923379-supplementary.pdf]

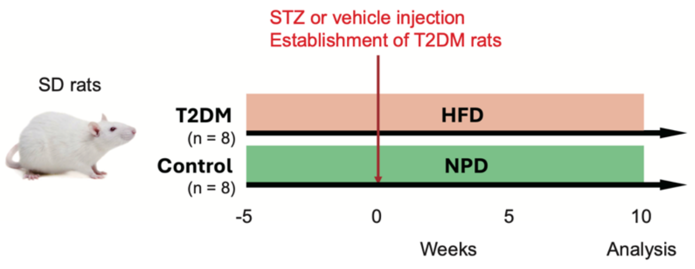

Figure S1. Study design scheme. The rats (n=16) were randomly divided into two dietary groups (n=8/group) via Microsoft Excel and fed either an NPD or an HFD ad libitum for the first 5 weeks. After the initial 5 weeks of dietary manipulation, the HFD-fed rats (DM group) were injected with low-dose STZ (35 mg/kg) intraperitoneally, while the NPD-fed rats (control group) were injected with vehicle citrate buffer.

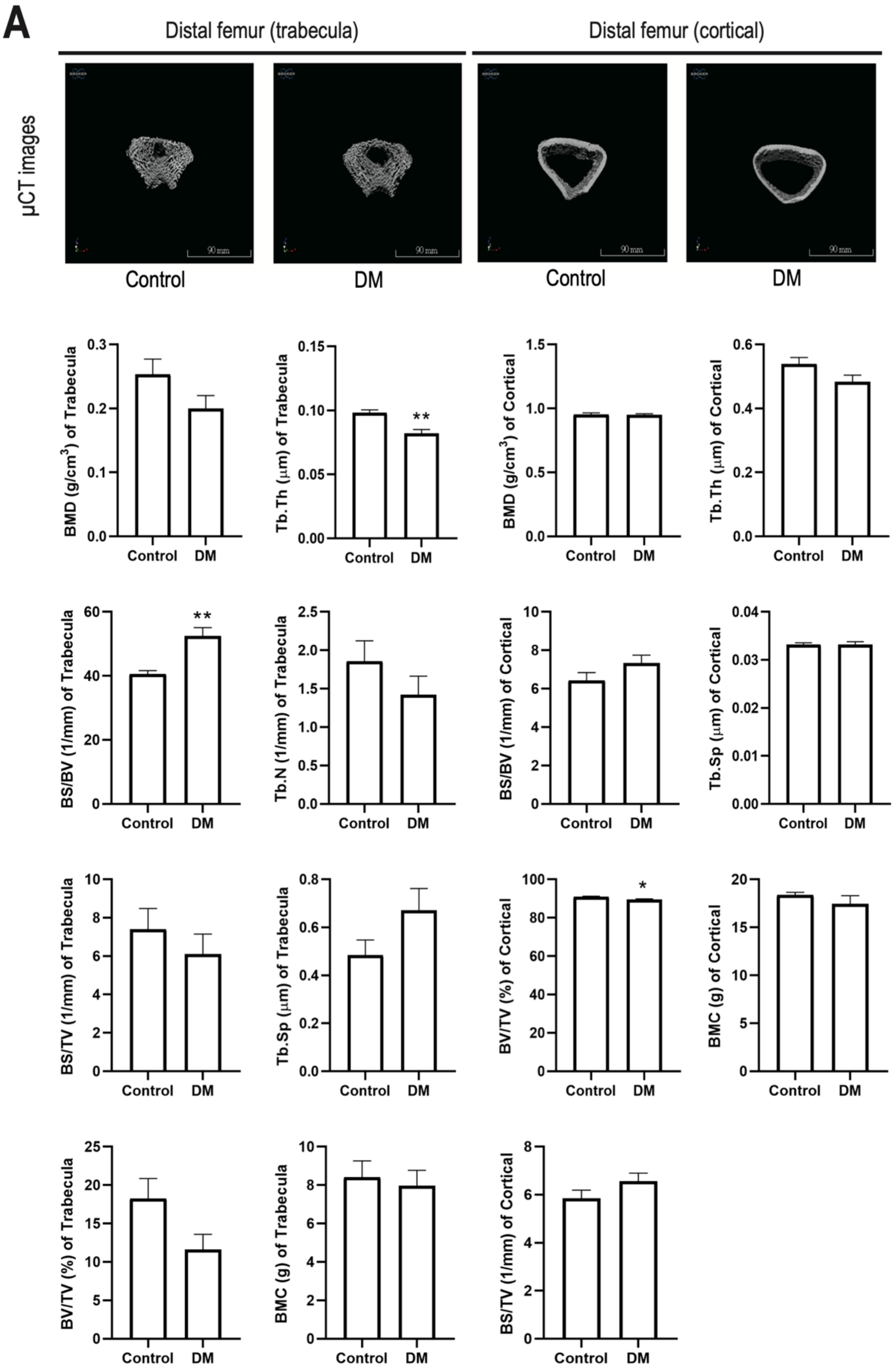

Figure S2. μCT parameters derived from the trabecular or cortical bone of the distal femur in control and DM groups. (A) Micro-computed tomography (**μ**CT) images were captured for the distal femur trabecular and cortical bone at week 18 for both the control and DM groups. Subsequently, bone parameters were analyzed using **μ**CT for the trabecular and cortical bone segments of the distal femur in each group. All data are presented as means ± SD (error bars) of 8 rats per group. Statistically significant differences were observed between the DM group and the control group at the same time point: * $P < 0.05$, ** $P < 0.01$, and *** $P < 0.001$ vs. control.

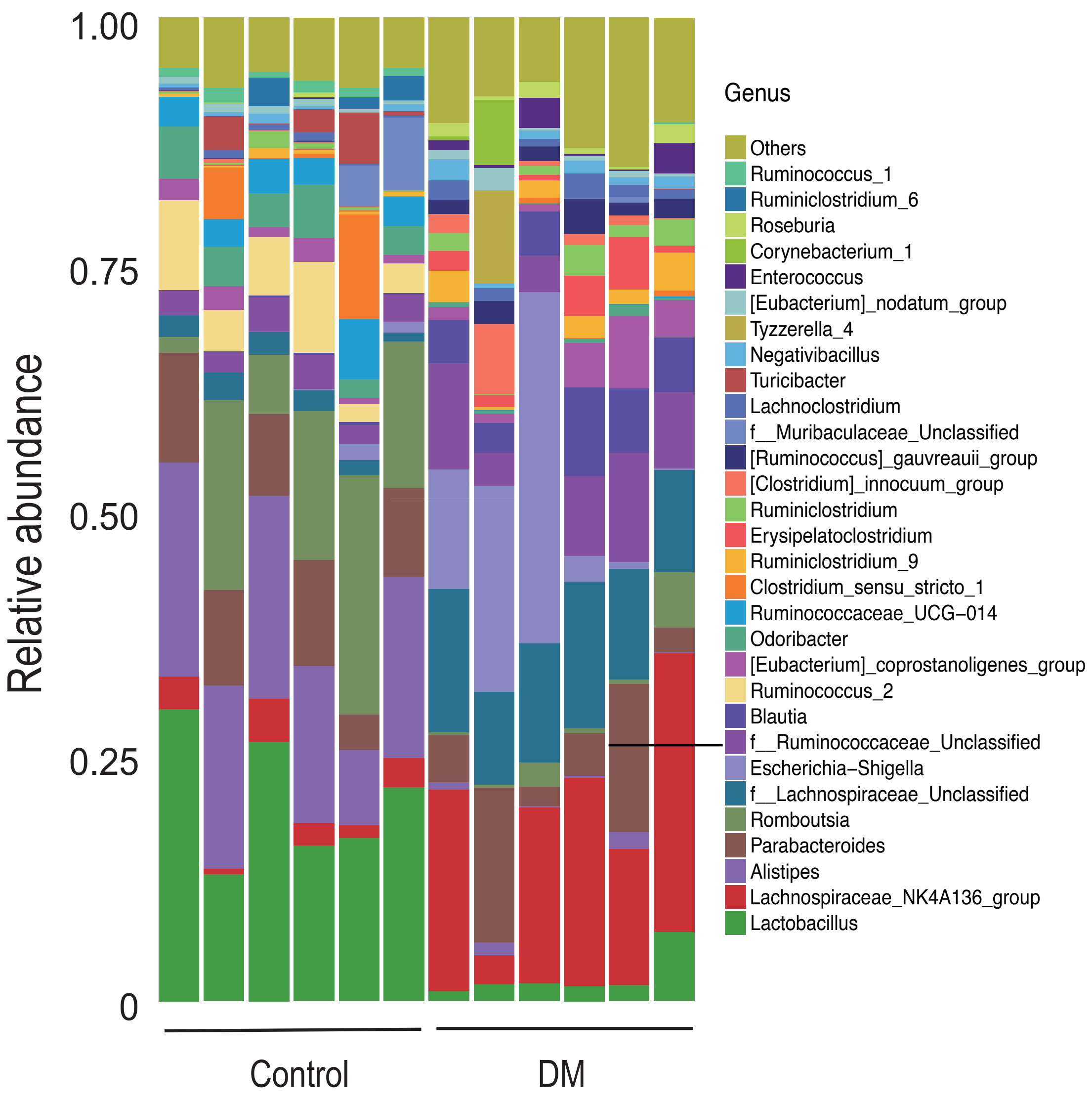

Figure S3. The magnified image of Figure 3A. Composition of microbiota at the genus level of the control and DM groups.

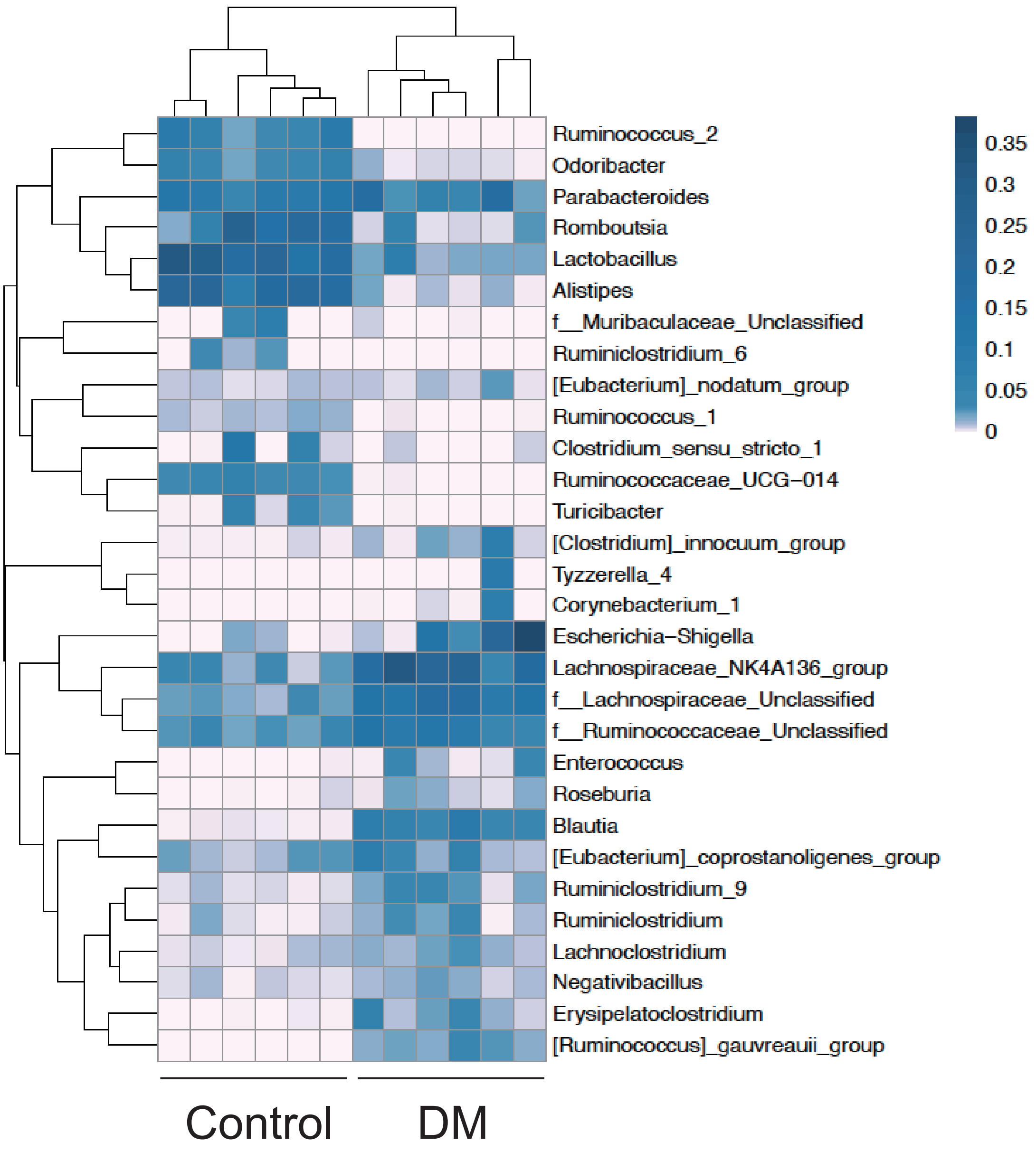

Figure S4. The magnified image of Figure 3B. A heatmap displaying the abundance information of the top 30 OTUs with the highest abundance, as well as the similarity and difference across OTUs and samples by similarity clustering.

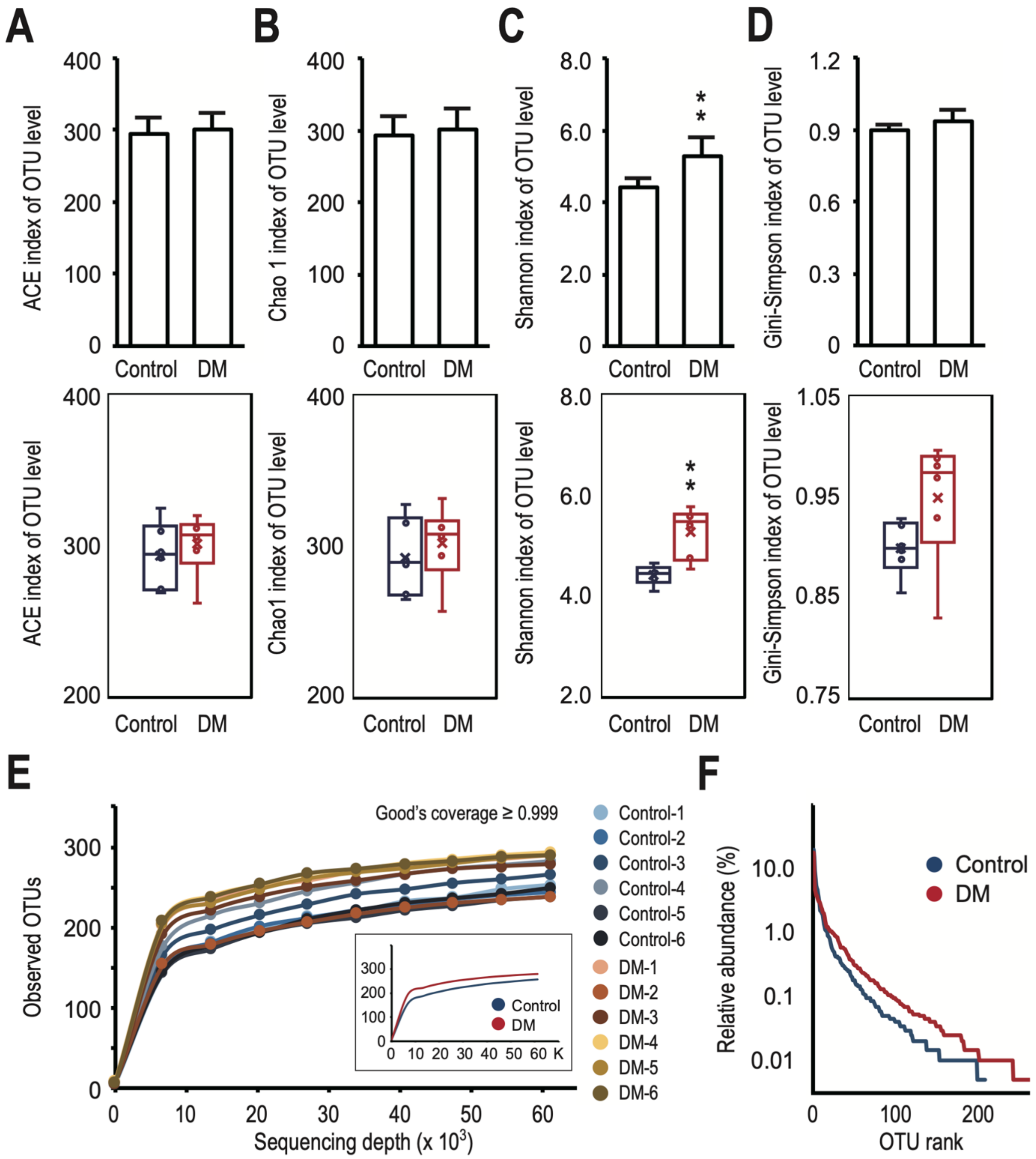

Figure S5. Comparison of gut microbiota diversity in control and T2DM rats. We evaluated alpha-diversity using ACE (A), Chao 1 (B), Shannon (C), and Gini-Simpson (D) indices to determine species richness, evenness, and dominance. In the top row, we've included bar charts that show means ± SD (error bars) of 6 rats per group. The bottom row includes box

and whisker plots, which show medians, upper/lower quartiles, and upper/lower extremes of 6 rats per group. We found that T2DM rats had higher species evenness without increased species dominance, but similar species richness compared with control rats (* $P < 0.05$, ** $P < 0.01$, and *** $P < 0.001$). To evaluate the number of operational taxonomic units (OTUs) observed in individual samples against sequencing depth, we created a rarefaction curve at 97% similarity level, as shown in (E). Samples were rarified at an even depth of 60,000 sequences per sample, and Good's coverage was higher than 0.999 for all samples. The inset shows the group-based rarefaction curve. Finally, we created a rank abundance curve by groups at 97% similarity level, as shown in (F). The horizontal axis displays the OTU abundance in rank order, and the vertical axis displays the relative abundance of sequences on a logarithmic scale.

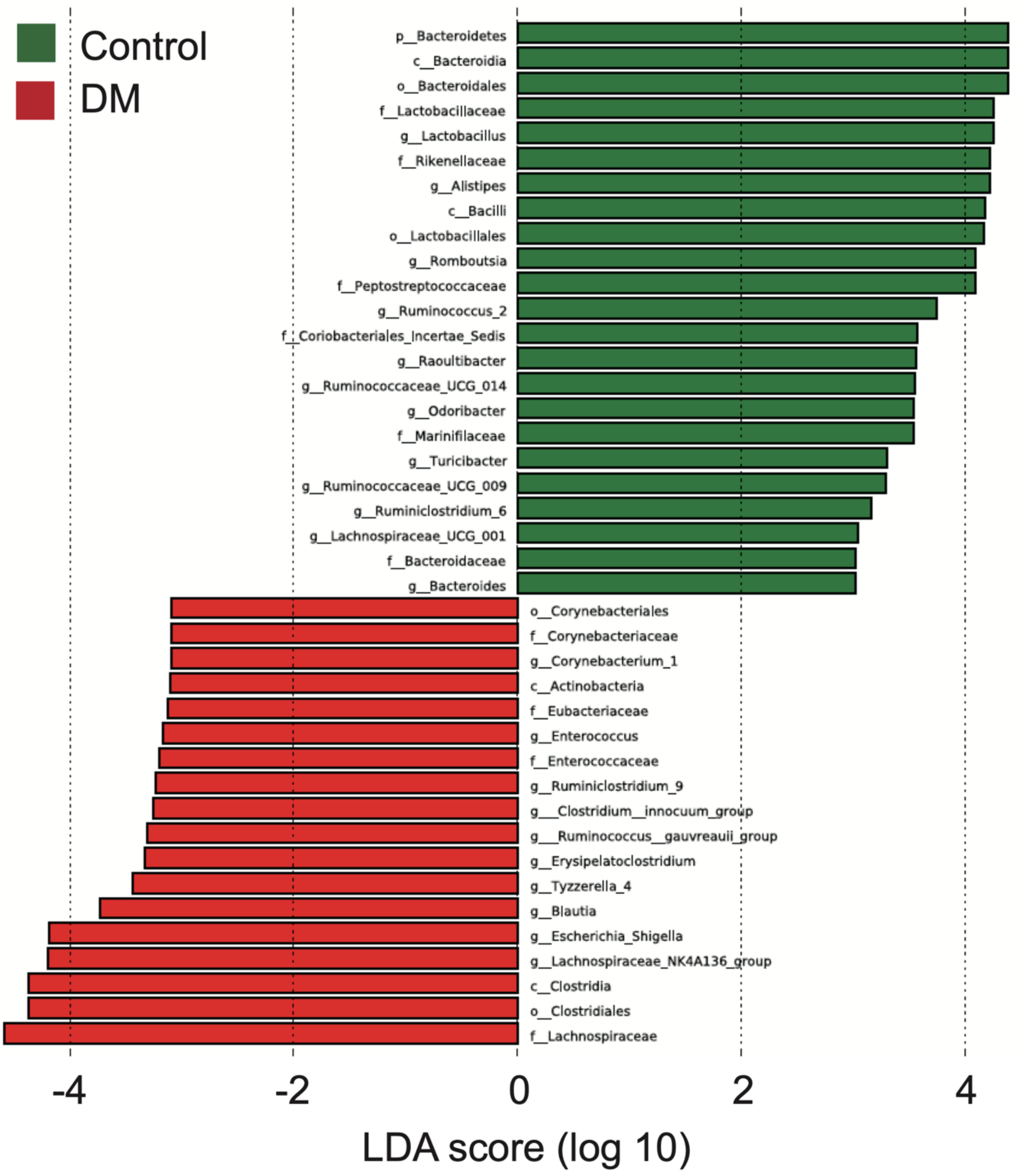

Figure S6. The magnified image of Figure 3I. A histogram of the linear discriminant analysis (LDA) scores presents species whose abundance showed significant differences between the control and DM groups.

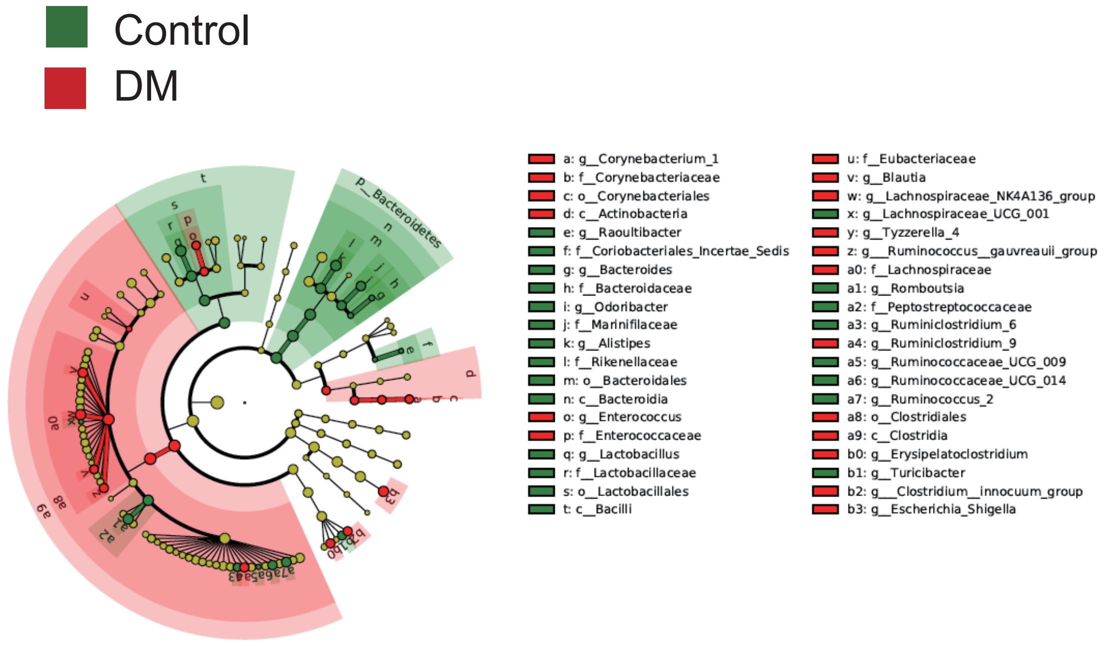

Figure S7. The magnified image of Figure 3J. A LEfSe (LDA effect size) cladogram was constructed to indicate the evolutionary relationships of different species.
